# COVID-19 self-testing, a way to “live side by side with the coronavirus”: Results from a qualitative study in Indonesia

**DOI:** 10.1371/journal.pgph.0000514

**Published:** 2022-10-21

**Authors:** Catherine Thomas, Sonjelle Shilton, Caroline Thomas, Claudius Mone Iye, Guillermo Z. Martínez-Pérez

**Affiliations:** 1 Peduli Hati Bangsa, Jakarta, Indonesia; 2 FIND, The Global Alliance for Diagnostics, Geneva, Switzerland; Institute of Development Studies, UNITED KINGDOM

## Abstract

Alongside mass vaccination for COVID-19, sustainable diagnostic strategies for SARS-CoV-2 are needed to empower local communities and help them complement health authorities’ efforts to end the pandemic in low- and middle-income countries. Indonesia is among the nations with an overstretched health system that may benefit from technological innovations, such as rapid SARS-CoV-2 antigen-detection tests for self-testing, to detect asymptomatic cases and interrupt the transmission of the virus to healthy individuals. In mid-2021, we conducted a qualitative research study with the aim of understanding key decision-makers’ values and preferences regarding the implementation of COVID-19 self-testing in Indonesia. This research received ethics approval from the Universitas Katolik Indonesia Atma Jaya and used a thematic analysis approach to explore the insights expressed by healthcare workers, representatives of civil society, and potential self-testing implementers in three geographies: Jakarta, Banten, and North Sulawesi. Thirty semi-structured interviews and six focus group discussions were carried out. As per the informants’ narratives, the Indonesian public might accept rapid SARS-CoV-2 antigen-detection self-testing as a tool that will enable them to test for COVID-19 at their own convenience. Concerns were expressed that the public might doubt the reliability of self-testing kits if these were not properly regulated and if counterfeit kits were known to be on the market. Fear of stigma, isolation, and clinical care costs were perceived to be among the drivers for self-test users to not report a reactive result. These fears might be mitigated, as per the informants’ opinions, by awareness raising, passing of regulations, and participatory engagement of a range of community actors, such as village officers. Decision-makers consider rapid SARS-CoV-2 antigen-detection self-testing to be a welcomed screening tool that could contribute to ensuring earlier access to treatment and decrease transmission of SARS-CoV-2 in Indonesia.

## Introduction

Coronavirus disease 2019 (COVID-19) is caused by the novel severe acute respiratory syndrome 2 virus (SARS-CoV-2), first identified in Wuhan, China, in December 2019 [1]. On 11 March 2020, the World Health Organization (WHO) declared COVID-19 a global pandemic [2]. Screening for COVID-19 cases at primary care-level is among the key public health actions recommended by WHO for the early detection of SARS-CoV-2-infected individuals, to help stop community transmission of SARS-CoV-2 [3]. Screening using rapid, point-of-care diagnostics allows the tracing of at-risk contacts, isolation of infected individuals, and accelerated access to treatment, if necessary [3,4]. In some countries, especially low- and middle-income countries (LMICs), primary care settings that lack laboratory capacity may face challenges in the use of point-of-care diagnostics and meeting the demand for COVID-19 testing.

Point-of-care diagnostics include portable real-time polymerase chain reaction (RT-PCR) machines [5], professional-use rapid SARS-CoV-2 antigen-detection tests (RATs) [6], and RATs for self-testing at home, school, or work [7,8]. Of these methods, RT-PCR is the most accurate; however, it is expensive, and it is dependent on the availability of trained personnel, as well as the optimal supply of reagents and specimen collection systems [5]. Professional RATs are a more affordable and deployable option than RT-PCR [6], although their utilization can also be difficult where there is a dearth of healthcare workers or where these workers are overburdened with their existing duties.

Indonesia is among those LMICs where the provision of professional mass screening to its entire territory, especially for populations living in hard-to-reach areas, is not feasible [9]. RATs for self-testing are devices that enable individuals to test for COVID-19 at their own convenience and without the assistance of a healthcare worker. RATs for self-testing can be used to complement health authorities’ testing efforts, as recommended by international organizations such as the European Union [10] and the African Union [10,11].

Research conducted in Indonesia has already demonstrated the acceptability of self-testing for human immunodeficiency virus (HIV) among men who purchase sex [12] and for hepatitis C virus (HCV) among people who are living with HIV and people who use injectable drugs [13]. Therefore, there is also the potential for the Indonesian public to accept rapid SARS-CoV-2 antigen-detection self-tests (hereafter referred to as “self-testing”). In Indonesia, self-testing could assist people who are unable to access facility-based testing to know whether they have COVID-19 and thus help prevent further transmission. However, similar acceptability research to that carried out for HIV and HCV self-testing is necessary to understand the Indonesian public’s values and preferences around self-testing.

Self-testing has not yet been broadly adopted in all LMICs. For example, as of January 2022, regulatory authorities in Brazil were discussing the regulation of self-tests for public use [14]. Further evidence is needed if LMICs are to uptake self-testing in a safe and effective way. The risk of false-negative results and of people not reporting a true-positive result out of fear of stigma, for instance, merit qualitative research to be conducted, to enable appropriate planning and introduction of self-testing in new sociocultural contexts. Mapping stakeholders’ views is a crucial step in informing the design of strategies for implementing self-testing. To tackle the current lack of evidence, we conducted the qualitative research reported here to assess decision-makers’ values in relation to SARS-CoV-2 self-testing to diagnose and prevent the spread of SARS-CoV-2.

## Methods

### Ethics statement

The survey protocol received ethical clearance from the Universitas Katolik Indonesia Atma Jaya (Reference: 0674A/III/LPPM-PM.10.05/06/2021). All informants provided written consent.

### Study design and sites

This qualitative followed the methodology proposed by Kielmann et al. [15] for conducting qualitative investigations in LMIC settings. The study involved semi-structured interviews and focus groups discussions as data generation methods. These methods were chosen with the aim of being able to compare how the decision-makers’ narratives diverged when they were interviewed individually in comparison with when they were interviewed in the company of other informants.

The research was conducted in three geographies within Indonesia (Jakarta, Banten province, and North Sulawesi), with the aim of obtaining representations from individuals exposed to a variety of sociocultural and economic environments. This qualitative research was conducted alongside a larger, population-based survey conducted in the same geographies, which had the goal of assessing values around, and the acceptability of, SARS-CoV-2 self-testing among the general public and the healthcare workforce [16].

### Study populations

The study included healthcare workers (HCWs) engaged in COVID-19 prevention and care; potential implementers (PIs) of self-testing programs; and representatives or spokespersons of civil society groups (RCSs). HCWs (e.g., nurses, physicians) were approached because of their capacity to recommend self-testing to their patients. PIs (e.g., leaders in private industries or large corporations, directors in departments of public health, etc.) were approached because of their capacity to pool and dedicate resources to the procurement and delivery of self-testing in the workplaces or geographies where they operate. RCSs (e.g., traditional community and religious leaders, representatives of trade unions or professional councils, etc.) were approached because of their capacity to influence community decision-making on the utility of self-testing. Eligibility criteria to participate in the study were that informants had be aged more than 18 years, they had to give informed consent, and they had to speak Bahasa Indonesia.

### Sampling procedures

The study team in Indonesia (principal investigator and two research assistants) sought potential informants using a variety of means (e.g., Google Maps, health facility registries, organizational registries, and websites of professional councils and academic environments) to prepare sex-disaggregated lists of at least 50 different potential informants per each of the three study populations. Efforts were made to ensure maximum variation of sampling in terms of gender identity and of urban and rural workplaces. To ensure diversity of institutional profiles in the sample, the study team did not invite more than one potential informant per clinic, community or institution of interest

To avoid convenience sampling and to attempt to mitigate the effect of social desirability [17], the sex-disaggregated lists prepared by the study team were randomly rearranged using the RANDOM.org randomizer. The interviewers contacted shortlisted informants by phone, starting with the first name on each list, and provided them with information about the study’s aim and procedures. Those who expressed an interest in the study were invited to participate in either a semi-structured interview or in a focus group discussion, but not both.

### Data collection

Depending on informant preferences, data collection was conducted using either Zoom teleconferencing software or in-person at a venue of both the informant and the interviewer’s choice.

The data collection was led, in the Bahasa Indonesia language, by a female and a male research assistant with backgrounds in the social sciences and experience in qualitative research. The same 45-question guide, administered in order, was used for both the semi-structured interviews and the focus group discussions (S1 Guide). The guide included questions based around six themes: knowledge of conventional, professional-led COVID-19 testing; values around the use of RATs for self-testing; the public’s preferences for the distribution and delivery of RATs for self-testing; aspects relating to how to encourage the safe and effective use of self-testing devices by the public; likely actions by members of the public upon receiving a reactive or a negative self-test result; and barriers to and recommendations for the distribution of self-tests in Indonesia (Supporting Information 1).

Respondents’ socio-demographic data were collected at the beginning of the semi-structured interviews and focus group discussions. All data collection encounters were audio-recorded. Recordings were transcribed verbatim by the research assistants who conducted data collection. Transcripts were cross-checked against the recordings for accuracy and completeness and then subsequently translated from Bahasa Indonesia into English. All translated transcripts were cross-checked against the Bahasa Indonesia transcripts.

### Data analysis

Transcripts were uploaded into Quirkos software, and a thematic analysis was applied by the study’s principal investigator, a female Chinese-Indonesian public health expert. We followed the guidance developed by Braun and Clarke [18] but mainly that developed by Kielmann et al. [15]. This approach was chosen as the most appropriate analytical methodology to identify those findings that could be used to inform policy development and public health practice.

Initially, all transcripts were deductively coded using a pre-defined coding scheme. Whenever an emerging theme was identified, a new code was inductively created. In parallel with the coding, reflexive memos were prepared to control for the risk of informant bias. Iteratively with the coding, the dataset was analyzed using a four-stage approach: transcript by transcript at first; followed by a theme-by-theme, sex-sensitive comparison of coded narratives across all transcripts, then by a theme-by-theme, rural- versus urban-sensitive comparison of coded narratives across all transcripts; and finally, a comparison of the key findings across the three study populations. Attention was paid to the memos to ensure that no informant biases from the analysts were introduced. COREQ guidelines were considered when reporting [19].

## Results

### Participants’ characteristics

A total of 53 decision-makers participated in 30 semi-structured interviews (Table 1) and 6 focus group discussions (Table 2). Nine RCSs, 11 HCWs, and 10 PIs were interviewed. Eight RCSs, eight HCWs, and seven PIs participated in focus group discussions (i.e., two focus group discussions were conducted per study population). Of the total, 27 self-identified as cis male individuals, and one self-identified as a trans female individual. Twenty-seven informants were based in Jakarta. The majority of informants (n = 39) had completed undergraduate university studies.

**Table 1:** Sociodemographic profile of the semi-structured interview participants.

No	Population	Location	Gender	Age	Education level; Profile
1	Representative of civil society	Rural	Female	27	Undergraduate; Muslim youth leader
2	Representative of civil society	Rural	Female	22	Undergraduate; teacher in non-formal education
3	Representative of civil society	Rural	Male	53	Undergraduate; Catholic men’s leader
4	Representative of civil society	Rural	Male	72	Undergraduate; leader of a teacher’s association
5	Representative of civil society	Rural	Female	28	Healthcare and housing coordinator for orphans and poor people
6	Representative of civil society	Urban	Female	36	Elementary school; program director of a non-governmental organization for victims of human trafficking
7	Healthcare worker	Urban	Male	39	Postgraduate; expert in occupational medicine
8	Representative of civil society	Urban	Male	38	Undergraduate; patient community leader
9	Representative of civil society	Urban	Male	46	Undergraduate; trade union leader
10	Representative of civil society	Urban	Female	40	Undergraduate; national coordinator of a sex worker network
11	Healthcare worker	Rural	Female	41	Undergraduate; physician
12	Healthcare worker	Rural	Male	29	Postgraduate candidate; physician
13	Healthcare worker	Rural	Male	51	Postgraduate; physician
14	Healthcare worker	Rural	Female	49	Undergraduate; head nurse
15	Healthcare worker	Rural	Female	48	Undergraduate; head of a communicable disease department at a community health center
16	Healthcare worker	Urban	Female	39	Undergraduate; clinic head
17	Healthcare worker	Urban	Female	39	Undergraduate; laboratory analyst
18	Healthcare worker	Urban	Male	37	Undergraduate; physician
19	Healthcare worker	Urban	Male	49	Undergraduate; physician
20	Healthcare worker	Urban	Female	37	Undergraduate; nurse
21	Potential self-testing implementer	Rural	Male	50	Undergraduate; telecommunication company general manager
22	Potential self-testing implementer	Rural	Female	47	Undergraduate; school principal
23	Potential self-testing implementer	Rural	Male	39	Undergraduate; owner of a tile distribution business
24	Potential self-testing implementer	Rural	Male	38	Senior high school; drug rehabilitation center program manager
25	Potential self-testing implementer	Rural	Female	40	Undergraduate; vice principal of an elementary school
26	Potential self-testing implementer	Urban	Female	38	Postgraduate; vice academic director of a university
27	Potential self-testing implementer	Urban	Female	49	Undergraduate; head of department at the Ministry of Health
28	Potential self-testing implementer	Urban	Male	34	Undergraduate; head of division at a Provincial Health Office
29	Potential self-testing implementer	Urban	Male	45	Graduate; operational director of a major Indonesian restaurant chain
30	Potential self-testing implementer	Urban	Male	41	Undergraduate; automobile branch manager

**Table 2:** Sociodemographic profile of the focus group discussion participants.

No	Population	Location	Gender	Age	Education level/Profile
1	Representative of civil society	Rural	Male	40	Senior high school; Buddhist community leader
2	Representative of civil society	Rural	Female	46	Undergraduate; area leader of a women’s empowerment and children’s protection agency
3	Representative of civil society	Rural	Female	49	Undergraduate; youth leader
4	Representative of civil society	Rural	Female	19	Senior high school; program manager of a writer’s community
5	Representative of civil society	Urban	Male	48	Undergraduate; founder of a non-governmental organization for patient care
6	Representative of civil society	Urban	Trans female	61	Undergraduate; chairperson of a non-governmental organization for trans women
7	Representative of civil society	Urban	Male	49	Undergraduate; head of a *Rukun Tetangga* (neighborhood association) in the north Jakarta area
8	Representative of civil society	Urban	Female	39	Senior high school; founder of a youth tuberculosis organization
9	Healthcare worker	Rural	Male	41	Postgraduate; orthopedic surgeon
10	Healthcare worker	Rural	Male	31	Undergraduate; hemodialysis nurse
11	Healthcare worker	Rural	Female	34	Undergraduate; emergency room nurse
12	Healthcare worker	Urban	Female	55	Postgraduate; psychologist
13	Healthcare worker	Urban	Male	36	Undergraduate; physician
14	Healthcare worker	Urban	Male	46	Undergraduate; pharmacist
15	Healthcare worker	Urban	Female	57	Undergraduate; midwife
16	Healthcare worker	Urban	Male	45	Undergraduate; physician
17	Potential self-testing implementer	Rural	Male	60	Undergraduate; area team leader of the COVID-19 Task Force
18	Potential self-testing implementer	Rural	Male	41	Undergraduate; Member of the House of Representatives
19	Potential self-testing implementer	Rural	Female	43	Undergraduate; owner of a machinery business
20	Potential self-testing implementer	Rural	Female	23	Undergraduate; regional manager of a lifestyle product retailer
21	Potential self-testing implementer	Urban	Male	45	Postgraduate; Board member of an Association of Indonesia Local Health Office
22	Potential self-testing implementer	Urban	Female	38	Undergraduate; owner of a home cleaning company
23	Potential self-testing implementer	Urban	Male	45	Undergraduate; bank manager

### Knowledge of COVID-19 detection and testing

In the informants’ opinion, many Indonesian people will seek a provider-initiated COVID-19 diagnosis when they travel, when they experience COVID-19-related symptoms, or when they have been in close contact with a case. When individuals need to test, the most accessible diagnostics for them are facility-based tests available in hospitals, laboratories, or clinics; train stations; and in the *Puskesmas* (i.e., government-funded primary-level healthcare centers).

Deterrents to the uptake of testing were identified. The fear of being forced by the healthcare system to isolate, which could limit their ability to earn an income, was described as a major deterrent. As one male PI from Jakarta noted, there are persons who are “grateful just being able to buy rice” and who may prefer to avoid isolation than to test.

A reason to refuse testing expressed by many informants was the “not that uncommon” belief that COVID-19 “does not exist”. No informant explained the origin of this belief.

Informants across all study geographies mentioned that another deterrent to testing that might be common to those who do not have the resources to use private testing services was related to health system-related inconveniences such as long queues, long turnaround times for results, limited testing hours and tests-per-day quotas, and the risk involved in encountering SARS-CoV-2-infected individuals in the clinics. In addition to these inconveniences described as possible reasons for not seeking a test, a few RCSs in North Sulawesi explained that some people living on the islands offshore would face difficulties in reaching testing sites.

Fears around stigma, “shame”, and death were mentioned among all groups as deterrents to testing that were important at the start of the pandemic. From the informants’ perspective, however, these fears were less prevalent in mid-2021 than they were during the early part of 2020, when having COVID-19 was similar to suffering “leprosy in the days of the Prophets”.

### Values around self-testing

Although some informants, including a few HCWs, thought that “COVID-19 self-testing” was a term that referred to people voluntarily seeking professional testing services and paying for them, the majority were aware of the availability of self-testing in Indonesia. The internet, social messaging groups, and the mass media were mentioned as common sources of information about self-testing.

Some informants reported having used self-testing. One male PI, a branch manager in Jakarta, showed the interviewer the saliva self-tests that he stocked at home for the “fifteen relatives” who were living in his compound. Another informant, a midwife from Jakarta, explained how she used rapid SARS-CoV-2 antigen-detection tests for home administration:

*“My boy had symptoms like coughing at night*, *having a cold*, *and he said ‘Please examine me’*. *And we used Lungene*. *I checked*, *and it was positive*.” Female, Midwife (HCW), Jakarta

The use of rapid SARS-CoV-2 antigen-detection tests for self-testing is a screening approach that was, according to some informants, endorsed by some healthcare professionals. As explained by a female nurse from Banten, some neighborhood officials in rural areas (e.g., youth leaders, village advisory officers) received training by *Puskesmas* on how to teach villagers to self-test and to report the results to their clinics. Another HCW, a male physician from Jakarta, opined that self-test devices are easy to use by lay people, without having to rely on health services. This opinion was shared by the majority of informants.

Most informants emphasized that self-testing would be beneficial to increase awareness of the disease and prevent further transmission of the virus. Self-testing would enable non-infected people to test in private without needing to go to a facility. A male RCS valued how self-testing is “the” solution to overcoming barriers to testing, as in the medium-term it will inevitably be integrated in people’s daily lives, because he perceived that society is moving toward accepting that everybody will need to live “side by side” with COVID-19.

Nevertheless, not all HCWs supported self-testing, mainly because of the possibility of invalid results. Other disadvantages identified by the HCWs included the challenges in identifying and monitoring of COVID-19 cases detected via self-testing. Difficulties for case tracing and the risk of false results were also mentioned by some PIs and RCSs, who also expressed doubts about the healthcare system’s preparedness to manage the increase in the number of COVID-19 cases that might occur if uptake of self-testing is generalized to all communities.

"*This self‑test might increase the number of false-negative results*. *Because the accuracy is very doubtful… because… the person who should have been positive*, *because of technical problems*, *ends up being negative*. *Maybe the transmission rate may also be higher because the person believes that their test result is negative but [the self-test is] technically inaccurate*.*"* Male, Physician (HCW), Jakarta

The informants believed that the beneficiaries of self-testing could be the general population and specific groups of people who are at increased risk of COVID-19 due to their personal vulnerabilities (e.g., comorbidities, being elderly) or to the nature of their occupation (e.g., students and teachers who are exposed to many different people on a daily basis). Nevertheless, the HCWs expressed that, for them to recommend self-testing, certain prerequisites should be met, such as the self-test kits must be used for screening and not to confirm a diagnosis; they must have high accuracy, be easy to use, and include user instructions in Bahasa Indonesia; and, they must be recognized by the Ministry of Health. Some PIs and RCSs also noted that, for them to recommend self-testing, the kits should be accurate, user-friendly, and endorsed by the Ministry of Health.

"*When a device or drug has not been approved by the Ministry of Health*, *I will not dare to recommend it*. *But when it is allowed*, *I will recommend it either to the individual*, *the community itself*, *or to a company*.*"* Male, Occupational Medicine Specialist (HCW), Jakarta

### Preferences for service delivery

According to most informants, the Indonesian public would prefer saliva self-tests, as these are perceived as easier, more comfortable, and less painful than blood or nasopharyngeal self-tests. Nasal swabs or finger-prick self-tests were described as “scary”, “traumatizing”, and complicated. Some PIs suggested that nasopharyngeal self-tests may increase the risk of user errors:

“*There are people whose swab tools have just touched the front of the nose and they won’t continue…I’m afraid they haven’t finished reading the instructions and the interpretation might be wrong*.” Male, Head of Hospital Division (PI), Jakarta

Despite these perceptions, a few RCSs and PIs expressed a preference for nasal swabs or blood self-tests, as they were perceived to be more accurate. Some HCWs reflected that any choice around self-tests should be based on the devices’ sensitivity and specificity rather than on the specimen required.

“*If the accuracy is the same with currently available antigen swab*, *and the price is cheaper*, *I highly recommend it*.” Male, Physician (HCW), North Sulawesi

There was agreement that self-tests could be made widely available at pharmacies, medical equipment stores, and at *Puskesmas* sites. A few informants also suggested that self-tests could be made available at schools, offices, or factories. Many rural RCSs suggested that self-tests could be available at neighborhood associations in any *Rukun Warga* (i.e., division of regions under the villages) or *Rukun Tetangga* (i.e., division of villages under *Rukun Warga*). A few PIs indicated that self-tests could be available at any public place, such as transport terminals, airports, or markets. One male PI opined that the distribution could follow the distribution line of consumer goods such as “Coca-Cola and cigarettes”, which was, according to him, “the widest and best-proven distribution chain”.

When discussing who the distributors could be, a concern about “counterfeit products” was raised by a few PIs. To prevent acquisition of “counterfeited” kits, or “fake” self-tests, some PIs expressed that they would not recommend buying self-tests from websites other than known “flagship stores” and those that receive official government permission to sell self-tests.

“*People are very creative with counterfeiting*. *Fake products*. *What needs to be anticipated is how to guarantee that the product in circulation is the original product*.” Female, Vice Principal (PI), Banten

Irrespective of where self-tests could be accessed, it was emphasized that they should be affordable, especially for socioeconomically-disadvantaged groups. There was no consensus on how much a device should cost. HCWs’ responses varied from Indonesian rupiah (IDR) 25,000 (1.76 United States Dollars (USD) to IDR 150,000 (10.57 USD), RCSs’ responses ranged from IDR 15,000 (1.05 USD) to IDR 500,000 (35.05 USD), and PIs’ responses ranged from IDR 10,000 (0.7 USD) to IDR 200,000 (14.02 USD).

The public’s preferred location for self-testing might be their own home. Some RCSs from Jakarta suggested that some individuals might prefer to self-test at their workplace, at transport terminals, or at markets. Nevertheless, some HCWs expressed that they would discourage self-testing in public spaces, as the self-testing procedure requires the removal of the mask.

“*If done in groups*, *in the end*, *instead of keeping the health protocol*, *we carry out risk behavior for transmission*. *Also*, *don’t do it in a narrow place*. *And*, *when there are roommates*, *for example*. *Or at the office*, *don’t hold it in meeting places*. *It’s better to stay in your own place*.” Male, Family Physician (HCW), Jakarta

Even if performed in their own homes, some end-users may need assistance from a third person. As explained by one trans female RCS from Jakarta, many transgender persons may be illiterate and would need assistance. Some RCSs and PIs thought that supervised home use of self-tests could be achieved with the aid of relatives, partners, or healthcare workers. Nevertheless, as with their refusal to accept that people should self-test in public spaces, some HCWs disagreed that self-testing should require any aid from persons who might then be unnecessarily exposed to the virus (i.e., in the event that the self-tester was infected with SARS-CoV-2).

### Supporting the safe use of self-testing

As the risk of invalid results was voiced as a likely impediment for the HCW to recommend self-testing, it was suggested that staff from either private pharmacies or *Puskesmas* clinics could teach end-users how to use self-tests. Correct usage could also be ensured, as per all study groups’ suggestions, through education provided by village officers at the *Rukun Tetangga-*level and by other “experts”, using a wide range of formats, including video tutorials uploaded to YouTube-like platforms.

User instructions should be written in non-complicated vocabulary, in a legible font size, and be accompanied by easy-to-interpret pictorials. User instructions must be inserted in each self-testing kit, and they could include barcoded links to online tutorials. User instructions should be clear about the kit’s accuracy, how to use it, how to read the result, and what its expiry date and “government license number” is. Some RCSs also expressed that the instructions should indicate the risks involved in self-testing, in reference to the possibility of invalid results. There was consensus that an explanation on what to do following the reading of the results should be a component of the instructions.

Reporting of a positive result could be done by going to any *Puskesmas* clinic, contacting the local COVID-19 Task Force, or using available reporting technology (e.g., hotlines, telemedicine systems, and tracing apps). A trans female RCS suggested that transgender individuals might report their results via trained, senior peer community members, while some PIs suggested that, if self-testing occurred in working environments, some people might choose to report a positive result to the health authorities with the aid of their employer.

“*Maybe you can use an application from the government that has been used*, *such as* Peduli Lindungi. *Report the results directly in the application*. *So*, *everyone who accesses the place can also be notified for the test*.” Female, Patient Group Leader (RCS), Jakarta

Despite their preference for facility-based post-test counseling only, a few HCWs discussed the role that the community-based *Tim Reaksi Cepat* (quick response teams) could play in ensuring linkage to care for those self-testing positive. Although technological aids such as telemedicine apps were suggested, a few urban RCSs noted that some of these already available aids are not always user-friendly.

“*Sometimes we get ping‑ponged at hotlines*. *We call but the person talking is not the person but the machine says*: *‘Thank you for calling the hotline center*. *If you want to consult*, *please press 1*.*’ Then*, *asked to continue*: *‘Press 6*.*’ [You] press 6 and later*: *‘Operator is busy*, *please wait*.*’*” Male, Trade Union Leader (RCS), Jakarta

To ensure linkage to care, some HCWs emphasized the importance of the users’ “honesty”, while some RCSs mentioned the users’ “conscience”. However, there were also informants in all groups who recognized that fear of isolating might be a more compelling factor than “honesty” and “conscience” for some asymptomatic individuals who, after receiving a positive result, might feel that they could manage the infection on their own.

### Taking action upon receiving a reactive self-test result for COVID-19

The psychosocial impact of a positive result may depend on the self-testers’ understanding of both the severity of COVID-19 disease and the use of self-testing as a screening (not diagnostic) tool. The informants indicated that people receiving a positive self-test result could be angry, in denial, worried about the result being false, or afraid of discrimination. The HCWs thought that pre-test counseling on what to do in the event of receiving a positive result would be crucial to mitigate any such potentially harmful effects. Some PIs emphasized that self-test users might be very concerned about how their workmates would react. One female PI from Banten suggested that the impact on the self-tester may be dependent on the level of awareness among individuals in their environment. This view was shared by one male PI from Jakarta, who considered that the impact in the workplace might be mitigated if there were no punitive measures in place against SARS-CoV-2-infected employees.

The same male PI stated that users who received a positive result may also “think positively” and self-isolate and inform their close contacts. Many RCSs also supported the idea that, if awareness was optimal, many self-testers would self-isolate by themselves and request support from their nearest facility or via the COVID-19 hotline. One HCW stated that a factor that might encourage people to report a positive result could be the need to obtain a medical clearance letter from their community leader:

“*If you want to go back to work*, *you must have a certificate of completion of isolation issued by the government*. *If you don’t have that letter*, *you are not being allowed to work*. *And*, *if they don’t report at the beginning*, *for example*, *and suddenly they get healthy and ask for the letter*, *the health center won’t be issuing the letter*: *‘We didn’t monitor you*, *why do you ask for an isolation completion letter*?*’*” Male, Occupational Medicine Specialist (HCW), Jakarta

Although the notion that uptake of recommended actions following a positive result would depend on the awareness of the users was commonplace, this notion was not shared by the informants who doubted that users would report a positive result due to financial worries. Even if users did not react “badly” to a positive result (e.g., feeling “depressed”), concerns about the costs of COVID-19 care or fear of losing their job might be impediment to report the result.

Some HCWs were concerned that upon receiving a positive result, some individuals, especially those who are asymptomatic, could forgo preventive measures such as self-isolation, mask wearing, and social distancing. In their opinion, awareness must be raised to better inform asymptomatic carriers of the risk of transmitting the virus to those individuals who are most vulnerable to suffering severe COVID-19 disease.

As with reporting the result, there were informants across all groups who mentioned that some individuals may not self-isolate due to economic concerns. As one informant put it: “They will die not because of COVID, but because they were hungry”. According to one RCS from Jakarta, in the transgender community self-isolation could be difficult due to the living conditions of some transgender persons:

“*For transgender friends whose home environment is not adequate*, *it is better not to stay at home for self-isolation*. *A mechanism must also be considered so that they can do self-isolation in a health facility if there are no severe symptoms*.” Trans female, Trans Persons Association Leader (RCS), Jakarta

Challenges for self-isolation were anticipated by most informants, even for users who would be willing to self-isolate. There was consensus that community support may increase uptake of isolation (e.g., some RCSs suggested providing individuals who were self-isolating three meals a day and contacting them via messaging apps or using telemedicine systems).

### Recommendations to overcome barriers to demand

Fear of “shame”, concerns about the kits’ authenticity and accuracy, as well as distribution issues (i.e., where to access the kits, especially for those living in rural areas or in the islands), were mentioned as barriers to demand that could be eliminated with good planning. It was suggested that HCWs be trained to teach people about self-tests; that self-tests be subsidized and made available at all *Puskesmas* sites; that the kits be packaged with proof of authenticity; and that a 24-hour hotline for pre- and post-test counselling be established. The PIs suggested that, to ensure wide distribution of self-tests, it could be helpful to capitalize on existing structures, such as local women’s and neighborhood associations, in addition to COVID-19 Task Forces.

It was suggested by HCWs and some PIs that *Badan Pengawas Obat dan Makanan* (BPOM) (i.e., the National Agency of Drug and Food Control of Indonesia) should issue regulations on self-testing and that these should be communicated to healthcare workers at all levels. The RCSs suggested that, should self-testing be available in pharmacies, that regulations on stockpiling, distribution permits, and medical waste management would be necessary.

Finally, barriers to demand could be tackled, according to some informants, if the Indonesian government officially encouraged serial self-testing. To facilitate serial self-testing, some HCWs suggested that self-tests be incorporated into screening programs at the lowest community administrative levels and be provided in schools and workplaces. Most PIs supported self-testing in the workplace. However, for at-work serial self-testing to be feasible, a few PIs indicated that regulations would be needed to guide their use by small-scale private companies. It was also noted that companies will need to enforce policies to protect people from being fired if they self-test positive.

## Discussion

In Indonesia, people seek provider-initiated COVID-**3**19 testing, but health service-related inconveniences (e.g., waiting times, stock-outs of rapid antigen-detection tests) are common deterrents for some people to test. Some individuals, including some of our informants, may procure rapid antigen-detection tests from e-commerce sites and self-administer them, although these tests had not been approved as self-tests by the Indonesian regulatory authority for health products (i.e., the *Badan Pengawas Odat dan Makanan*) when we conducted our study.

In this context, our study shows that SARS-CoV-2 self-testing, if endorsed by health authorities and distributed alongside awareness-raising and pre- and post-test counseling, could represent an easy to use, and convenient solution that would be beneficial in identifying asymptomatic carriers, initiating isolation and contact tracing, and, ultimately, decreasing the transmission of SARS-CoV-2. This is in line with findings from a cross-sectional survey in Indonesia, which found that 62.7% of people agreed with the concept of individuals being able to self-test for COVID-19 [13].

There was no consensus among our informants about how self-testing should be made available or about how self-test users might behave following a positive result. In this regard, all groups agreed that provisions are needed to ensure that the self-tests the population has access to are authentic and accurate. The issue of having a self-test recognized or legitimized by the *Badan Pengawas Odat dan Makanan* or the Ministry of Health was described in this study as being crucial to gain public trust. Indeed, counterfeit products in Indonesia range from cosmetics, food and beverages, software, leather goods, clothing, and even drugs, causing a loss to the national economy of IDR 65.1 trillion in 2014 [20]. Ensuring public trust in self-testing will require a substantial degree of effort, education, information, and community mobilization by health authorities.

An aspect that was voiced by some informants was that the impact of self-testing on pandemic control will be dependent on public health stakeholders’ capacity to create awareness targeted at fueling societal support for self-testers. Self-test users will need to be trusted as much as the self-testing devices they use. There is a need to sensitize healthcare workers, representatives of civil society groups, and implementers in public institutions and private corporations. Sensitization efforts must emphasize the requirement to not take punitive measures against self-testers who might be perceived as not being capable of making the best use of this testing approach. Our informants identified myriad factors that may contribute to individuals’ hesitancy to undertake COVID-19 testing in general, which may also extend to their actions upon receiving a positive self-test result, such as reluctance to isolate, to warn their contacts or employers, or to report a positive self-test result. Factors identified related to contextual and structural impediments, such as poverty, hunger, and job precariousness, which will not be easy to overcome in the short-term.

Some HCW informants in our study perceived that there may be the possibility of user errors, challenges in case reporting, and psychosocial ills following a positive result. While psychosocial harm following notification of a positive COVID-19 test result is also frequent in high-income contexts [21], the under-reporting of positive results has already been documented in Indonesia [22]. It follows from this that the distribution of self-tests cannot achieve a successful outcome without educating the public. Education about self-tests, as well as case monitoring, was recommended by our informants to be performed in the conventional way (e.g., through *Puskesmas* or neighborhood leaders) and also through the use of telemedicine systems. Currently, a telemedicine website and the Ministry of Health website have warnings for the public about the risks of using a self-test at home [23,24]. With the rise of telemedicine systems in Indonesia, several free apps have been made available for self-isolating patients [25].

Self-testing may become the “norm” if the epidemiological landscape changes and Indonesians begin to think that they will need to start living alongside COVID-19. Globally, self-testing is becoming more accepted and more widely used. The surge in the Omicron variant of concern has caused many governments to start pondering how to live alongside COVID-19, leading to countries such as Peru [26] and China [27] finally approving self-testing for public use. It should be noted that, in our study, narratives about the social and economic impact of the pandemic held more weight in the informants’ appreciation of self-testing as a decentralized screening approach than narratives around the morbidity and mortality caused by COVID-19. There was unanimity in terms of making COVID-19 self-testing kits as affordable as possible, which is aligned with African Union [11] and WHO [28] guidance on self-testing. Financial shocks during the pandemic caused people to prioritize their basic needs over COVID-19 testing and isolation, even in the presence of symptoms. One report suggested that, under the most extreme scenario, the poverty rate in Indonesia will increase to 12.4%, indicating that more than 8.5 million people in Indonesia will become impoverished [29]. Public health measures cannot be effective if people’s ability to meet their basic needs are not considered.

A key recommendation arising from this study is the need to consider community-based agents, such as neighborhood groups, youth groups, and village officials, as essential decision-makers who should be involved in the rollout of self-testing. These agents know first-hand the difficulties faced by the people they support. Due to their empathy, therefore, they could become important front-line actors in the support of the use of self-testing and in the event of receiving a positive result. In practice, *Puskesmas* clinics may be considered crucial, as they play a coordinating role between the communities and public health authorities.

Our study has some limitations. The informants were recruited from different geographies, and we pursued diversity with regard to gender identities, location, and socio-professional and institutional profiles. However, this was a qualitative inquiry, and our decision-makers’ perspectives may not be representative of all possible opinions in Indonesia. Our findings offer insights that might be characteristic of the specific groups represented in our study. A strength is that we ensured balance in terms of gender representation; however, a more in-depth gender analysis of the informants’ narratives using validated sex- and gender-based analytical approaches [30,31] would be necessary in any future self-testing implementation programs, to better understand what the impact of this screening approach might be across gender groups in Indonesia. Additionally, it must be noted that social desirability bias may have occurred, as this is commonplace in qualitative research involving group discussions. Also, some data collection encounters were performed via teleconferencing software. The content of encounters conducted online and in-person was not different; however, it must be acknowledged that it is easier to build rapport with interviewees during face-to-face encounters. A final concern is that, with the availability of professional rapid antigen-detection tests via e-commerce sites in Indonesia, the informants might have conflated insights into the self-administration of professional rapid antigen-detection tests and devices marketed as self-tests for the general public as identical approaches. During the data analysis process, it was often difficult to discern whether the informants were referring to provider-administered or self-administered rapid SARS-CoV-2 antigen-detection tests. To avoid the introduction of observant bias from our side, the narratives of a few informants around their perceived advantages and values of self-testing could not be considered.

In conclusion, this qualitative study involved different profiles of decision-makers who would be involved in the recommendation, distribution, and implementation of SARS-CoV-2 self-testing in Indonesia. These individuals described how the Indonesian public might accept this screening approach, not only to test for SARS-COV-2 at their own convenience and hence know their status but also to protect their loved ones, stop the onward transmission of the virus to other individuals, and help them make decisions about how to continue with their routine economic activities and not jeopardize their household’s economy, in ways that would not create further risks for the community. With a proper regulatory framework, training of healthcare workers, and engagement of communities, the decision-makers involved in this study considered SARS-CoV-2 self-testing a screening tool to be welcomed, which could contribute to increasing uptake of testing and to decreasing the transmission of SARS-CoV-2 in Indonesia.

## Supporting information

S1 Guide Individual interviews and focus group discussions guide. (DOCX)
