# Supplementary material for: COVID-19 self-testing, a way to “live side by side with the coronavirus”: Results from a qualitative study in Indonesia
Source: PLOS Glob Public Health. 2022 Oct 21;2(10):e0000514. doi: 10.1371/journal.pgph.0000514 (PMC10021662; doi:10.1371/journal.pgph.0000514)
Supplement: S1 Guide — (DOCX) [file pgph.0000514.s001.docx]

Supporting Information

S1. Individual Interviews and Focus Group Discussions Guide

| THEME 1: Knowledge and experience with conventional COVID-19 testing | |
| --- | --- |
| Q1. Do you know how COVID-19 manifests? | *Local names of the disease; Sources of knowledge; Awareness of implications of being in a pandemic; Signs and symptoms; different manifestations in high vulnerability vs low vulnerability groups; perceptions of degree of morbidity / mortality* |
| Q2. Do you know how transmission of COVID-19 can be prevented? | *Virus; Vectors and fomites; Risk practices; Factors than increase opportunities for contagion; Awareness of one’s own perception of risk; Hygiene and prevention means; barriers and facilitators of prevention* |
| Q3. What should people do to find out if they have COVID-19? | *People at-risk; Triggers of demand of COVID-19 detection; duration between recognition of the symptoms and demanding a test; Venues and institutions where to demand COVID-19 detection* |
| Q4. When COVID-19 diagnosis is needed, how can it be done? | *Rapid tests; RT-PCR; Immunoassays; Clinic vs. hospital-based diagnostics; home-based kit deliveries; Antigen vs. antibody assays* |
| Q5. May you describe the COVID-19 diagnostic services that you know that are available for the population? | *Volunteer vs. qualified provider-based testing; Hospital, Facility & Community-based, home-based; Agents testing for COVID-19 (e.g. Nurses, lab technicians, physicians, community health worker); Costs* |
| Q6. Are you engaged in COVID-19 testing? | *Tell me about how you as a healthcare provider / community representative / implementer are engaged in testing...* |
| Q7. Who are the users of these existing COVID-19 diagnostics? | *General population; travellers; At-risk populations; Healthcare workers; People demanding testing vs. people invited/forced to test; Voluntary testing vs. being referred to testing by a healthcare work* |
| Q8. What do you think are the reasons why some people do not go for COVID-19 testing? | *Stigma; Discrimination; Costs and payments; Fear (to the disease, to the healthcare workers…); lack of access/availability; distrust of the health systems; painful procedure; symptoms are indisputable/no need to test; fear of being forced into isolation/ quarantine. Inefficient link to COVID-19 care and treatment; Time and geographical availability; Quality of healthcare provision...* |
| Q9. What do you think are the reasons why healthcare workers do not reach all people who should receive a COVID-19 testing? | *Enacted discrimination; lack of resources, technology, staff; Lack of training and capacities; Inter-professional conflicts; Poor screening strategies…; different perceptions of who should/shouldn’t get tested; lowering perceived population-level prevalence rates by decreasing diagnosis rates* |
| THEME 2: Value of COVID-19 Self-Testing | |
| Q10. Have you ever heard of people testing for COVID-19 by themselves? | Knowledge of what a self-test is; Sources of knowledge; Previous experience |
| Q11. A self-test kit similar to the one commonly used for pregnancy is proposed. What could be the advantages of allowing people to use it? | Public health; Elimination of COVID-19; Prevent transmission; Timely initiation into treatment |
| Q12. And do you see any disadvantages? | Psychosocial harm; Consequences of receiving a false negative or a false positive; non-disclosure of COVID-19 status; implications for epidemiological surveillance and accurate estimations of prevalence, implications for public health, More expensive than facility-based testing; Poor linkage to care |
| Q13. What type of people could be interested in self-testing for COVID-19? | *Other at-risk groups; Groups who would refuse its use; Differences men/women, young/old, etc.* |
| Q14. As a healthcare staff / community leader / implementer: Will you recommend COVID-19 self-testing to the population? | *Previous use; Usefulness; Ease of use; Ease of understanding; User errors...* |
| THEME 3: General Population’s Preferences for Service Delivery  Interviewer READS before each question: *“If COVID-19 self-testing were available to the general population…”* | |
| Q15. ...what type of specimen should COVID-19 self-testing request for people to accept it? | *Blood, urine, saliva…; nasal / throat swab* |
| Q16. ...what should be its maximum price for people to be willing to purchase it? | *Free-of-charge for certain populations; Populations who could afford and/or would be willing to buy self-tests; Financial problems; Concept of public health system* |
| Q17. ...what should be its accuracy or precision for people to trust it? | *Accuracy; User errors; Trust in one’s capacities* |
| Q18. …where should it be accessible? | *Pharmacy; Kiosk; Lab; Clinic; Association; From peers; Internet; Partner-delivered; Vending machines; At the workplace...* |
| Q19. ...who should be authorized to distribute or give self-testing? | Healthcare workers; vendors at convenience stores or supermarkets, online |
| Q20. ...what type of information should be in the self-testing kit? | *Literacy issues; Cognitive problems; Learning problems; Disabilities; Lack of privacy and intimacy; audiovisual guides, online tutorials, hotline for questions* |
| Q21. ...where would people prefer to use it? | *Supervised vs. unsupervised; Home vs. clinic* |
| Q22. ...with whom would people prefer to use it? | intimate partner; parent; other family members; friends; healthcare worker; by themselves |
| Q23. ...if they needed help, from whom would they accept counselling and advice? | *Post-counselling; Police and judicial (i.e. in case of suffering gender-based violence, facing a partner/employer forcing them to self-test, etc.); Peer-educator; Support to read results, telephone (hotline) assistance,* |
| Q24. ...how should people receive an explanation on how to link to COVID-19 treatment after its use? | hotline, online linkage using QR code or other code, displacement to the health facility, kit-contained protocol upon receiving a positive and negative result |
| THEME 4: Safe and Effective Use of COVID-19 ST  Interviewer READS before each question: *“If COVID-19 self-testing were available…”* | |
| Q25. Under what circumstances do you think that COVID-19 self-testing should not be distributed/provided? | General opinion |
| And, if self-testing were to become available, how do you think it should be provided... | |
| Q26. ...so that the most vulnerable or stigmatized are not left behind? | *Differences between general and vulnerable groups…; Differences between men/women, young/adults, rural/urban…* |
| Q27. ...to ensure correct use? | *Differences between general and vulnerable groups…; Differences between men/women, young/adults, rural/urban…* |
| Q28. ...to ensure accurate results? | *Differences between general and vulnerable groups…; Differences between men/women, young/adults, rural/urban…* |
| Q29. ...to ensure linkage to COVID-19 care? | *Differences between general and vulnerable groups…; Differences between men/women, young/adults, rural/urban…* |
| Q30. ...to ensure quarantine/isolation? | *Differences between general and vulnerable groups…; Differences between men/women, young/adults, rural/urban…* |
| Q31. ...to ensure contact tracing? | *Differences between general and vulnerable groups…; Differences between men/women, young/adults, rural/urban…* |
| THEME 5: Taking Action Upon a COVID-19 ST-reactive RESULT | |
| Q32. If a self-test is positive, how do you think people would react? | *Differences between general and vulnerable groups…; Differences between men/women, young/adults, rural/urban…* |
| Q33. Do you think that they would communicate it to their nearest clinic? | *barriers and facilitators; implications of communicating / not communicating it to the clinic* |
| Q34. Do you think that they would start using hygienic and preventive measures? | *barriers and facilitators; implications* |
| Q35. Do you think that they would self-isolate? | *barriers and facilitators; implications* |
| Q36. Do you think that they would warn the people with whom they have been in touch? | *barriers and facilitators; implications* |
| Q37. If a self-test is reactive, what could be the psychosocial impact in the person using the self-test? | *denial; fear of stigma; considerations of non-disclosure; false positive; factors that could reduce social harm* |
| THEME 6: Future Prospects | |
| Q38. What would be the biggest barriers people could have to access COVID-19 self-testing? | *At-risk populations; Discrimination; Time and financial constraints; lack of availabity; living in a remote location; fear of being labelled a COVID-19 suspect* |
| Q39. What can we do to minimize or impede all those difficulties to access COVID-19 self-testing? | *Social welfare; Financial support; Counselling; Social Change; Research; Advocacy; Training health personnel...* |
| Q40. What type of policy and regulatory changes will be needed in your environment to facilitate that people access COVID-19 self-testing? | *recognition of ST as a valid diagnostic tool; free provision of ST* |
| Q41. What type of improvements in public health practice will be needed in your environment to facilitate that people access and use COVID-19 self-testing? | *training healthcare workers on pre- and post ST counselling, wider availability, free of charge ST provision* |
| Q42. What type of capacity building will be needed in your environment to facilitate that healthcare workers accept and promote COVID-19 self-testing? | *pre- post-test counselling, recognition of the validity of the ST, algorithms, patient flows* |
| Q43. What type of community sensitization and mobilization will be needed in your environment to facilitate correct understanding of COVID-19 self-testing? | Advertisements, outreach, social media, community leaders, other stakeholders |
| Q44. What type of measures will be needed in your environment to facilitate implementation of serial and regular testing using COVID-19 self-testing kits? | Procedures, norms; Organizational culture; Patient and employees rights; Promotion of serial testing; Linkage to care |
| Q45. What are your final recommendations so that the community accepts COVID-19 self-testing? | Final recommendations, appraisal of the study and its procedures |
